# Acute dose of melatonin via Nrf2 dependently prevents acute ethanol-induced neurotoxicity in the developing rodent brain

**DOI:** 10.1186/s12974-018-1157-x

**Published:** 2018-04-21

**Authors:** Tahir Ali, Shafiq Ur Rehman, Fawad Ali Shah, Myeong Ok Kim

**Affiliations:** 1 Division of Applied Life Science (BK 21), College of Natural Sciences, Gyeongsang National University, Jinju, 660-701 Republic of Korea; 2 Department of Pharmacology, Riphah Institute of Pharmaceutical Sciences, Riphah International University, Islamabad, Pakistan

**Keywords:** Melatonin, Ethanol, Neurotoxicity, ROS/oxidative stress, Nuclear factor erythroid 2-related factor 2 (Nrf2), p-NF-_K_-B/p-IKKβ pathway, MAPK-p-P38-JNK pathway, Neuroinflammation, Neurodegeneration

## Abstract

**Background:**

Melatonin is a well-known potent endogenous antioxidant pharmacological agent with significant neuroprotective actions. Here in the current study, we explored the nuclear factor erythroid 2-related factor 2 (Nrf2) gene-dependent antioxidant mechanism underlying the neuroprotective effects of the acute melatonin against acute ethanol-induced elevated reactive oxygen species (ROS)-mediated neuroinflammation and neurodegeneration in the developing rodent brain.

**Methods:**

In vivo rat pups were co-treated with a single dose of acute ethanol (5 g/kg, subcutaneous (S.C.)) and a single dose of acute melatonin (20 mg/kg, intraperitoneal (I.P.)). Four hours after a single S.C. and I.P. injections, all of the rat pups were sacrificed for further biochemical (Western blotting, ROS- assay, LPO-assay, and immunohistochemical) analyses. In order to corroborate the in vivo results, we used the in vitro murine-hippocampal HT22 and microglial BV2 cells, which were subjected to knockdown with small interfering RNA (siRNA) of Nrf2 genes and exposed with melatonin (100 μM) and ethanol (100 mM) and proceed for further biochemical analyses.

**Results:**

Our biochemical, immunohistochemical, and immunofluorescence results demonstrate that acute melatonin significantly upregulated the master endogenous antioxidant Nrf2 and heme oxygenase-1, consequently reversing the acute ethanol-induced elevated ROS and oxidative stress in the developing rodent brain, and in the murine-hippocampal HT22 and microglial BV2 cells. In addition, acute melatonin subsequently reduced the activated MAPK-p-P38-JNK pathways and attenuated neuroinflammation by decreasing the expression of activated gliosis and downregulated the p-NF-_K_-B/p-IKKβ pathway and decreased the expression levels of other inflammatory markers in the developing rodent brain and BV2 cells. Of note, melatonin acted through the Nrf2-dependent mechanism to attenuate neuronal apoptosis in the postnatal rodent brain and HT22 cells. Immunohistofluorescence results also showed that melatonin prevented ethanol-induced neurodegeneration in the developing rodent brain. The in vitro results indicated that melatonin induced neuroprotection via Nrf2-dependent manner and reduced ethanol-induced neurotoxicity.

**Conclusions:**

The pleiotropic and potent neuroprotective antioxidant characteristics of melatonin, together with our in vivo and in vitro findings, suppose that acute melatonin could be beneficial to prevent and combat the acute ethanol-induced neurotoxic effects, such as elevated ROS, neuroinflammation, and neurodegeneration in the developing rodent brain.

## Background

Increasing evidence indicates that ethanol exposure can cause a major insult to the central nervous system (CNS) that induces detrimental effects in the developing brain and is a leading cause of birth defects, mental retardation, and neurodevelopmental disorders. Maternal exposure to ethanol causes fetal alcohol syndrome (FAS), which is characterized by many neurological disorders, including cognitive and behavioral impairments. Acute administration of ethanol to postnatal pups produces remarkable neurodegenerative effects, and studies have shown that the neurotoxicity that is induced in the developing brain may persist for a long time, even into adulthood [1–4]. Several mechanisms have been investigated and proposed for ethanol-induced neuronal degeneration; the well-documented mechanism is the generation of elevated reactive oxygen species (ROS), which increase oxidative stress and consequently lead to the development of neurological disorders. Ethanol affects the actions of various endogenous antioxidant systems in the brain because the blood-brain barrier is permeable to ethanol and the normal homeostasis system of the brain becomes dysregulated [5, 6]. The CNS is one of the most sensitive organs to oxidative stress due to its high consumption of oxygen and limited number of antioxidant enzyme systems. Several mechanistic approaches are required to counteract the ethanol-induced, ROS-mediated detrimental effects in the developing brain. Notably, the upregulation of nuclear factor erythroid 2-related factor 2 (Nrf2), a key and master endogenous antioxidant gene, plays a key role against ROS-induced oxidative stress. Upregulated Nrf2 activates other endogenous redox-regulated enzymes, such as heme oxygenase-1 (HO-1) and glutathione cysteine ligase modulatory subunit (GCLM), consequently counteracting the ROS-induced oxidative stress and providing beneficial effects in various diseases, particularly neurodegenerative diseases [7–9].

Melatonin, also known as *N*-acetyl-5-methoxytryptamine, is a well-known potent endogenous antioxidant pharmacological agent and functions as a direct free oxygen radical scavenger and an indirect antioxidant; therefore, melatonin mitigates the detrimental effects and toxicity that are induced by elevated ROS and oxidative stress [10–13]. A large body of evidence has shown that melatonin can be used to treat pediatric patients with respiratory distress syndrome, bronchopulmonary dysphasia, and seizure disorders and to abrogate oxidative stress during sepsis and asphyxia [14–18]. Melatonin is a well-known neuroprotective agent against several neurodegenerative insults in the neonatal rat brain [19, 20]. Yon et al. reported that melatonin abrogated the severity of anesthesia-induced apoptotic neurodegeneration in the developing rat brain [21]. Melatonin attenuated brain injury induced by systemic lipopolysaccharide exposure in the postnatal rat brain [22]. Other studies have also demonstrated the beneficial effects of melatonin on fetal growth in response to any insults during pregnancy [15, 23, 24]. Recently, it has been reported that melatonin supplementation attenuated neuronal tube dysfunction in neonates during a diabetic pregnancy [25]. Hence, given the diverse neuroprotective properties that are associated with melatonin and considering that melatonin is a pleiotropic pharmacological agent and a potent neuroprotective antioxidant in nature, we hypothesized that acute melatonin would stimulate the master endogenous antioxidant Nrf2/HO-1 pathway and would ameliorate acute ethanol-induced detrimental effects, such as elevated ROS, neuroinflammation, and neurodegeneration in the developing rat brain.

## Methods

### Chemicals

Absolute ethanol (99.99%), melatonin, dimethyl sulfoxide (DMSO) and 2′7′-dichlorodihydrofluorescein diacetate (DCFH-DA) were purchased from Sigma Chemical Co. (St. Louis, MO, USA).

### Rat pups grouping and treatment

**Fig. 1:**
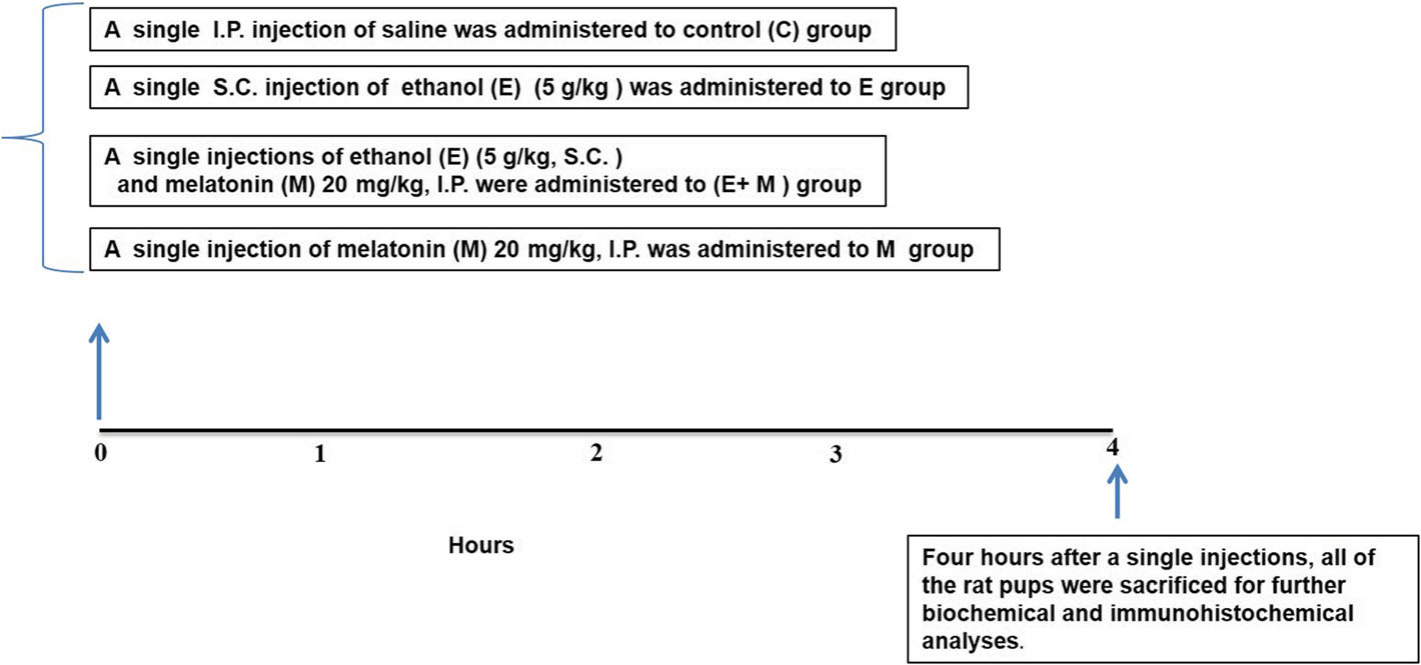
Schematic representation of the in vivo study design. Randomly selected male and female Sprague-Dawley postnatal day 7 rat pups of average body weight (18 g) were used (*n* = 15 pups/group). The postnatal rats were divided into the following groups: (1) Rat pups treated with a single intraperitoneal (I.P.) injection of saline as a vehicle, grouped as control (C). (2) Rat pups treated with a single dose of ethanol (E) (5 g/kg, subcutaneous (S.C.)), grouped as E. (3) Rat pups co-treated with a single dose of ethanol (5 g/kg, (S.C.)) and a single dose of melatonin (20 mg/kg, I.P.), grouped as E + M. (4) Rat pups treated with a single dose of melatonin (M) 20 mg/kg, I.P., grouped as M. Four hours after a single S.C. and I.P. injections, all of the rat pups were sacrificed for further biochemical and immunohistochemical analyses

Randomly selected male and female Sprague-Dawley postnatal day 7 rat pups of average body weight (15–18 g) were used (*n* = 15 pups/group). The experimental procedures were approved by the animal ethics committee of the Division of Applied Life Sciences, Department of Biology at Gyeongsang National University, Republic of Korea. Figure 1 describes the grouping and treatment of postnatal rats, which were divided into the following groups:

(1) Rat pups treated with a single intraperitoneal (I.P.) injection of saline as a vehicle, grouped as control (C)

(2) Rat pups treated with a single dose of ethanol (E) (5 g/kg, subcutaneous (S.C.)), grouped as E

(3) Rat pups co-treated with a single dose of ethanol (5 g/kg, (S.C.)) and a single dose of melatonin (20 mg/kg, I.P.), grouped as E + M

(4) Rat pups treated with a single dose of melatonin (M) 20 mg/kg, I.P., grouped as M

Four hours after a single S.C. and I.P. injections, all of the rat pups were sacrificed for further biochemical and immunohistochemical analyses. Melatonin was first dissolved in 0.1% DMSO and then in saline to reach the final administered volume. After fundamental and primary studies in the postnatal rat, we choose and proceeded to use the optimized acute dose of melatonin 20 mg/kg, I.P.; other studies also used this acute dose of melatonin [21].

### Protein extraction from rat pup brain tissue for the Western blotting analysis

Four hours after a single S.C. and I.P. injections, the rat pups (*n* = 10 rat pups per group) were sacrificed and the brains were immediately removed, and hippocampal tissues were dissected carefully and frozen on dry ice and stored at − 80 °C. The hippocampal tissues were homogenized in pro-prep™ protein extraction solution according to the manufacturer’s instructions (iNtRON, Biotechnology, Inc). However, we separated the cytosolic and nuclear fractions using a nuclear and cytoplasmic protein extraction kit, according to the manufacturer’s instruction (catalog # K266-25) Biovision Incorporated, A 95035 USA. The samples were then centrifuged at 10000×*g* rpm at 4 °C for 25 min. The supernatants were collected and stored at − 80 °C.

### Protein extraction from rat pup brain tissue for the ROS and LPO assays

A separate cohort of animals were used for the ROS and LPO assays. Four hours after a single S.C. and I.P. injections, the rat pups (*n* = 10 rat pups per group) were sacrificed and the brains were immediately removed and stored at − 80°C. The brain tissues were homogenized in the pro-prep™ protein extraction solution according to the manufacturer’s instructions (iNtRON, Biotechnology, Inc). The homogenized were then centrifuged at 10000×*g* rpm at 4 °C for 25 min (min). The supernatants were collected and stored at − 80°C until processed for the ROS and LPO assays.

### In vivo and in vitro ROS analysis

An in vivo ROS assay was performed to determine the level of ROS based on the oxidation of DCFH-DA to 2′7′-dichlorofluorescein (DCF) as described previously [26]. The conversion of DCFH-DA to DCF was analyzed using a spectrofluorimeter with excitation at 484 nm and emission at 530 nm. For background fluorescence (conversion of DCFH-DA in the absence of homogenate), we analyzed parallel blanks. To evaluate the elevated ROS, the results were analyzed and calculated as relative DCF pmol/mg protein.

Further, we also analyzed in vitro intracellular ROS in the mouse hippocampal (HT22) neuronal cells (a kind gift from Prof. Koh, Gyeongsang National University, Republic of Korea) via the DCF fluorescence intensity using confocal microscopy. The HT22 neuronal cells were cultured in 75-cm^2^ flasks (Thermo scientific, Nunc™ EasYFlask™ 75 cm2 Nunclon™ Delta surface, thermofisher scientific A/S, Kamstrupvej 90.P.O.Box 280 DK-4000 Rosklide, Denmark) containing Dulbecco’s modified Eagle medium (DMEM) (Gibco by life technologies, Grand Island, NY, USA) supplemented with 10% fetal bovine serum (FBS) and 1% antibiotics (penicillin-streptomycin) at 37 °C in humidified air containing 5% CO_2_. The number of HT22 cells was counted using a disposable hemocytometer (the full grid on a hemocytometer contained 9 squares of 1 square mm each) through the addition of 10 μl of the media that contained the cells to both sides of the hemocytometer chamber. Four 1/25 square mm corners and the middle central square were used to count the cells under × 10 magnification using Olympus IX70 microscope. The average number of cells in both sides of the chamber was calculated, and the cells were further subcultured in (2 × 10^4^/ml) chamber slides (Thermofisher Scientific 75 Panorama Creek Drive Rochester, NY14625–2385, USA) in DMEM supplemented with 10% FBS and 1% antibiotics (penicillin-streptomycin) at 37 °C in humidified air containing 5% CO_2_. After the cells reached 70–80% confluence, they were treated with ethanol (100 mM), co-treated with ethanol (100 mM) and melatonin (100 μM); cells in the control group were treated with DMSO (0.01%). After 12 h, the cells were washed with 0.01 M phosphate buffer saline (PBS), followed by the addition of 50 μM DCFH-DA in DMSO and incubation at 37 °C in humidified air containing 5% CO_2_ for 30 min covered by aluminum foil. Afterward, the cells were washed with 0.01 M PBS and fixed in 4% paraformaldehyde. The slides were mounted with 4′, 6′-diamidino-2-phenylindole (DAPI) and Prolong Antifade Reagent (Molecular Probe, Eugene, OR), and the images were captured using laser confocal FluoView FV 1000 microscope equipped with FV10-ASW 3.1 Viewer (Olympus, Tokyo, Japan). The number of original confocal images per well of the chamber slide was five per group and the images were converted into tagged image file format (TIF) images. The fluorescence intensity of the same region of TIF images for all groups was measured using ImageJ software (National Institutes of Health, Bethesda, MD) via the following method. The TIF image background was optimized according to the threshold intensity and analyzes the immunofluorescence intensity at the same threshold intensity for all groups, and was expressed as the relative integrated density of the samples relative to control cells.

### Lipid peroxidation (LPO) analysis

The LPO levels were determined in the brain homogenates via the analysis of the level of malondialdehyde (MDA), a biomarker of LPO, using the thiobarbituric acid reactive substance (TBARS) assay, which was performed according to the manufacturer’s instructions (catalog # K739-100) from Biovision Incorporated, A 95035 USA). Absorbance at 535 and 520 nm was measured using a spectrophotometer. The TBARS concentration was determined using the standard 1, 1, 3, 3- tetra ethoxy propane (TEP), and the results were analyzed and calculated as relative MDA nmol/mg protein.

### In vitro culture and Nrf2 gene silencing by small interfering RNA (siRNA)

Murine microglia (BV2) cells (a kind gift from Dr. I. W. Choi, Inje University, Busan, Republic of Korea) and HT22 neuronal cells were cultured in 75-cm^2^ flasks (Thermo scientific, Nunc™ EasYFlask™ 75 cm^2^ Nunclon™ Delta surface, thermo fisher scientific A/S, Kamstrupvej 90.P.O.Box 280 DK-4000 Rosklide, Denmark), and the number of BV2 and HT22 cells were counted using a disposable hemocytometer (the full grid on a hemocytometer contained 9 squares of 1 square mm each) through the addition of 10 μl of the media that contained the cells to both sides of the hemocytometer chamber. Four 1/25 square mm corners and the middle central square were used to count the cells under × 10 magnification using Olympus IX70 microscope. The average number of cells in both sides of the chamber was calculated. The cells (2 × 10^4^/ml) were further subcultured in 35-mm Petri dishes (Thermo scientific, Nunc A/S, Kamstrupvej 90.P.O.Box 280 DK-4000 Rosklide, Denmark) in DMEM supplemented with 10% FBS and 1% antibiotics at 37 °C in humidified air containing 5% CO_2_. The Nrf2 expression in cells was knocked down with the Nrf2 gene-silencing siRNA at a concentration of 10 μM per transfection for 36 h according to the manufacturer’s protocol (SC: 37049, Santa Cruz Biotechnology, Inc.). Negative siRNA (Ambion, USA) was used as a control. The transfection was performed with lipofectamine™ 2000 reagent (Invitrogen) when the culture was 75–80% confluent. After 36 h of transfection, the cells were treated with ethanol (100 mM), co-treated with ethanol (100 mM) and melatonin (100 μM). Cells in the control group were treated with DMSO (0.01%). After 12 h, the cells were further processed for western blot analysis.

### In vitro ROS assay using HT-22 and BV2 cells with Nrf2 siRNA

The HT-22 neuronal and BV2 cells were cultured in 75-cm^2^ flasks (Thermo scientific, Nunc™ EasYFlask™ 75 cm2 Nunclon™ Delta surface, thermo fisher scientific A/S, Kamstrupvej 90.P.O.Box 280 DK-4000 Rosklide, Denmark) containing DMEM supplemented with 10% FBS and 1% antibiotics at 37 °C in humidified air containing 5% CO2, and the number of HT22 and BV2 cells were counted using a disposable hemocytometer (the full grid on a hemocytometer contained 9 squares of 1 square mm each) through the addition of 10 μl of the media that contained the cells to both sides of the hemocytometer chamber. Four 1/25 square mm corners and the middle central square were used to count the cells under × 10 magnification using Olympus IX70 microscope. The average number of cells in both sides of the chamber was calculated. The cells (2 × 10^4^/ml) were further subcultured cultured in 96 well plate (Thermofisher Scientific 75 Panorama Creek Drive Rochester, NY14625-2385, USA) containing DMEM supplemented with 10% FBS and 1% antibiotics at 37 °C in humidified air containing 5% CO2. After the cells reached 70% confluence, they were transfected with 10 μM Nrf2 siRNA with lipofectamine™ 2000 reagent for 36 h according to the manufacturer’s protocol. Negative siRNA was used as a control. After 36 h of transfection, the cells were co-treated with ethanol (100 mM) and melatonin (100 μM) for 12 h. After 12 h of treatment, the cells were exposed to 50 μM DCFH-DA and incubated for 30 min. The relative absorbance of the ROS-positive cells treated with DCFH-DA was measured at 488/530 nm with a multidetector (Promega).

### Western blot analysis

The protein concentration for both in vivo and in vitro was measured using the BioRad protein assay kit, BioRad Laboratories, CA, USA. Equal amounts of protein (15–30 μg) were electrophoresis using 4–12% Bolt™ Mini Gels (Novex, Life Technologies, Kiryat Shmona, Israel). The membranes were blocked in 5% (*w*/*v*) skim milk to reduce non-specific binding and incubated with primary antibodies (anti-Nrf2, anti- HO-1, anti-ionized calcium binding adapter molecule 1 (Iba-1), anti-phosphorylated-nuclear factor kappa B (p-NF-_K_B) 65, anti-phospho-c-Jun N-terminal kinase 1 [p-JNK1] (T183/Y185) p-JNK, anti-total-JNK, anti-tumor necrosis factor-α (TNF-α), anti-interleukin-1β (IL-1β), anti-nitric oxide synthase-2 (NOS-2), anti-cyclooxygenase-2 (COX-2), anti-caspase-3, anti-poly (ADP-ribose) polymerase-1(PARP-1) (PARP-1), anti-glial fibrillary acidic protein (GFAP), anti-cytochrome c (Cyt.c), anti-Bax, anti-Bcl2 and anti-β-actin from Santa Cruz Biotechnology, Dallas, TX, USA; p-IKKβ, anti-mitogen activated protein kinase phosphorylated-P38 (MAPKp-P38) and anti-total-P38 from Cell Signaling Technology, Beverly MA, USA; and anti-GCLM from abcam (discover more) overnight at 4 °C at a 1:1000 dilution. After reaction with a horseradish peroxidase (HRP)-conjugated secondary antibody, as appropriate, the proteins were detected using an ECL detection reagent according to the manufacturer’s instructions (Amersham Pharmecia Biotech, Uppsala, Sweden). The X-ray films were scanned, and the optical densities of the bands were analyzed through densitometry using the computer-based Sigma Gel program, version 1.0 (SPSS, Chicago, IL, USA).

### Enzyme linked immunosorbent assay (ELISA) using in vivo and in vitro samples

The developing rat brain homogenates and the in vitro BV2 cell lysates of the above experimental groups proceeded for the NF-KBP65 ELISA analysis (Life Technologies) according to the manufacturer’s suggested protocols.

### Brain sections preparation from rat pups for morphological analysis

Four hours after a single S.C. and I.P. injections, the rats (*n* = 5 rat pups per group) were perfused transcardially with 4% ice-cold paraformaldehyde, and the brains were post-fixed for 72 h in 4% paraformaldehyde and transferred to 20% sucrose for 72 h. The brains were frozen in O.C.T. compound (A.O, USA), and 14-μm coronal sections were cut using a CM 3050C cryostat (Leica, Germany). The sections were thaw-mounted on probe-on plus charged slides (Fisher, USA).

### Confocal microscopy analysis for single and double immunofluorescence

Confocal microscopy was performed for single and double immunofluorescence as described previously with some modification [26]. In brief, slides containing brain sections were washed twice for 10 min each in 0.01 M PBS, 1X proteinase-k was added to the tissue, and the slides were incubated at room temperature for 5 min. The slides were washed twice for 5 min each, followed by incubation for 1 h in blocking solution containing 2% normal serum and 0.3% Triton X-100 in 0.01 M PBS according to the antibody treatment. After the slides were blocked, they were incubated overnight at 4iC in the primary antibodies (Nrf2, p-JNK, GFAP, TNF-α, NOS-2, caspase-3, Iba-1, from Santa Cruz Biotechnology, Dallas, TX, USA, MAPKp-P38 and p-NF-_K_B65 from Cell Signaling Technology, Beverly, MA, USA, mouse monoclonal 8-Oxoguanine (8-OxoG) from Millipore, Billerica, MA, USA) diluted 1:100 in blocking solution. Following incubation with primary antibodies, the sections were incubated for 2 h in the secondary tetramethyl rhodamine isothiocyanate (TRITC)/fluorescein isothiocyanate (FITC)-labeled antibodies (1:50) (Santa Cruz Biotechnology). After the incubation with the TRITC/FITC-labeled antibodies, the sections were incubated overnight in another primary antibody, followed by an incubation with FITC/TRITC-labeled antibodies (1:50) under the same conditions. The slides were mounted with DAPI and Prolong Antifade Reagent. The images were captured using laser confocal FluoView FV 1000 microscope equipped with FV10-ASW 3.1 Viewer (Olympus, Tokyo, Japan). The number of original confocal images per tissue was five per group and the images were converted into TIF images. The fluorescence intensity of the same region of the cortex/total area and hippocampus/total area of the TIF images for all groups was measured using ImageJ software via the following method. The TIF image background was optimized according to the threshold intensity and analyzes the immunofluorescence intensity at the same threshold intensity for all groups and was expressed as the relative integrated density of the samples relative to the control.

### Immunohistochemical analysis

The immunohistochemical assay was performed according to our previous described protocol with minor modification [27]. The slides were washed twice for 10 min in 0.01 M PBS, followed by quenching for 10 min in a solution of methanol containing 30% hydrogen peroxidase and incubated for 1 h in blocking solution containing 5% normal goat serum and 0.3% Triton X-100 in 0.01 M PBS. After blocking, the slides were incubated overnight in rabbit anti-Nrf2 and mouse ant-HO-1 diluted 1:100 in blocking solution. Following incubation with primary antibody, the sections were incubated for 1 h in biotinylated goat anti-rabbit and rabbit anti-goat secondary antibody diluted 1:500 in 0.01 M PBS and subsequently incubated with ABC reagents (Standard VECTASTAIN ABC Elite Kit; Vector Laboratories, Burlingame, CA) for 1 h in the dark at room temperature. The sections were washed twice with PBS and incubated in 3, 3′-diaminobenzidine tetra hydrochloride (DAB) (Sigma, St. Louis, MO, USA) substrate. The sections were washed with distilled water, dehydrated in graded ethanol (70%, 95% and 100%), placed in xylene and coverslipped using mounting medium. Immunohistochemical TIF images were captured with a fluorescence light microscope. The number of images per slide was five per group. The immunohistochemical intensity for the number of nuclear Nrf2 and HO-1 activated cells in the hippocampus (CA1 region)/total area and cortex/total area of the brain were counted using the ImageJ software via the following method. The TIF image background was optimized according to the threshold intensity and analyzes intensity for the number of nuclear Nrf2 and HO-1 activated cells at the same threshold intensity for all groups and was expressed as the relative integrated density for the number of nuclear Nrf2 and HO-1 activated cells/section of the samples relative to the control.

### Terminal deoxynucleotidyl transferase dUTP nick end labeling (TUNEL) immunohistochemical staining

The TUNEL immunohistochemical assay was performed according to our previous described protocol [28]. Immunohistochemical TIF images were captured with a fluorescence light microscope. The number of images per slide was five per group. The inmmunohistochemical intensity for the number of activated TUNEL neuronal cells in the hippocampus (CA1 region)/total area and cortex/total area of the brain were counted using the ImageJ software via the following method. The TIF image background was optimized according to the threshold intensity and analyzes intensity for the number of the number of TUNEL positive neuronal cells at the same threshold intensity for all groups and was expressed as the relative integrated density for the number of TUNEL neuronal cells/ section of the samples relative to the control.

### Fluoro-jade B (FJB) immunohistofluorescence staining

The FJB assay was performed according to our previous described protocol with minor modification [29]. Briefly, the slides containing brain tissue were air-dried overnight. The slides were washed twice with 0.01 M PBS for 5 min. Following washing, the slides were immersed in a 1% sodium hydroxide and 80% ethanol solution for 5 min and then in 70% alcohol for 2 min followed by 2 min in distilled water. Next, the slides were transferred to a solution of 0.06% potassium permanganate for 10 min over a slow shaker, rinsed with distilled water, and then immersed in a 0.1% acetic acid and 0.01% FJB solution for 20 min. The slides were then rinsed with distilled water and dried for at least 10 min. The slides were mounted with DAPI and glass cover slips using DPX non-fluorescent mounting medium. The representative images were captured using a FITC filter on a confocal laser-scanning FluoView FV 1000 microscope equipped with FV10-ASW 3.1 Viewer (Olympus, Tokyo, Japan). The number of original confocal images per tissue was five per group, and the images were converted into TIF images. The inmmunohistochemical intensity for the number of activated FJB positive neuronal cells in the hippocampus (CA1 region)/total area and cortex/total area of the TIF images for all groups were measured using ImageJ software via the following method. The TIF image background was optimized according to the threshold intensity and analyzes the FJB positive neuronal cells at the same threshold intensity for all groups and was expressed as the relative integrated density for the number of FJB neuronal cells/section of the samples relative to the control.

### Nissl immunohistochemical staining

Nissl staining was used for the histomorphological evaluation of neuronal loss. The slides with 14-μm sections were washed twice for 15 min each in 0.01 M PBS and were stained with a 0.5% cresyl violet solution (containing a few drops glacial acetic acid) for 10–15 min. The sections were washed with distilled water and dehydrated in graded ethanol (70%, 95% and 100%), placed in xylene and coverslipped using mounting medium. Immunohistochemical TIF images were captured with a fluorescence light microscope. The number of images per slide was five per group. The inmmunohistochemical intensity for the number of dead, fragmented and shrunken neuronal cells in the hippocampus/total area (CA1 and DG region) and cortex/total area of the brain were counted using the ImageJ software via the following method. The TIF image background was optimized according to the threshold intensity and analyzes the dead, fragmented and shrunken neuronal cells at the same threshold intensity for all groups and was expressed as the relative integrated density for the number of dead neuronal cells/section of the samples relative to the control.

### ApoTox-Glo™ Triplex assay in HT22 cells using Nrf2 siRNA

ApoTox-Glo™ Triplex assay (Promega Corporation, 2800 Woods Hollow Road Madison, WI53711–5399, USA) was performed to assess viability, cytotoxicity and caspase-3/7 activation in the HT22 cells using Nrf2 siRNA. HT22 cells (2×10^4^/ml) were cultured in 96 well plates containing DMEM supplemented with 10% FBS and 1% antibiotics at 37 °C in humidified air containing 5% CO2. After 70% confluences the cells were transfected by Nrf2 siRNA 10 μM per transfection with lipofectamine™ reagent 2000 for 36 h according to the manufacturer protocol. The negative siRNA was used as control. After 36 h of cell transfection, the cells were co-treated with ethanol (100 mM) and melatonin (100 μM) for 12 h. After completion of the specified time, the cells were further preceded and the assay was performed according to our previous described protocol [28].

### Data analyses and statistics

The western blot bands were scanned and analyzed through densitometry using the Sigma Gel System (SPSS Inc., Chicago, IL). One-way analysis of variance (ANOVA) followed by a two-tailed independent Student’s *t* test and Tukey’s multiple comparison test were used for comparisons among the treated groups and the control. The Image-J software (National Institutes of Health, Bethesda, MD) was used for immunohistological quantitative analysis. The histograms and graphs were generated using GraphPad Prism 5 (GraphPad Software, San Diego, CA). The density values of the data were expressed as the means ± SEM of three independent experiments. *P* values less than 0.05 were considered to be statistically significant.

## Results

### Acute melatonin attenuated the acute ethanol-induced increase in ROS and oxidative stress in the rat pups as well in HT22 and BV2 cells exposed to ethanol

**Fig. 2:**
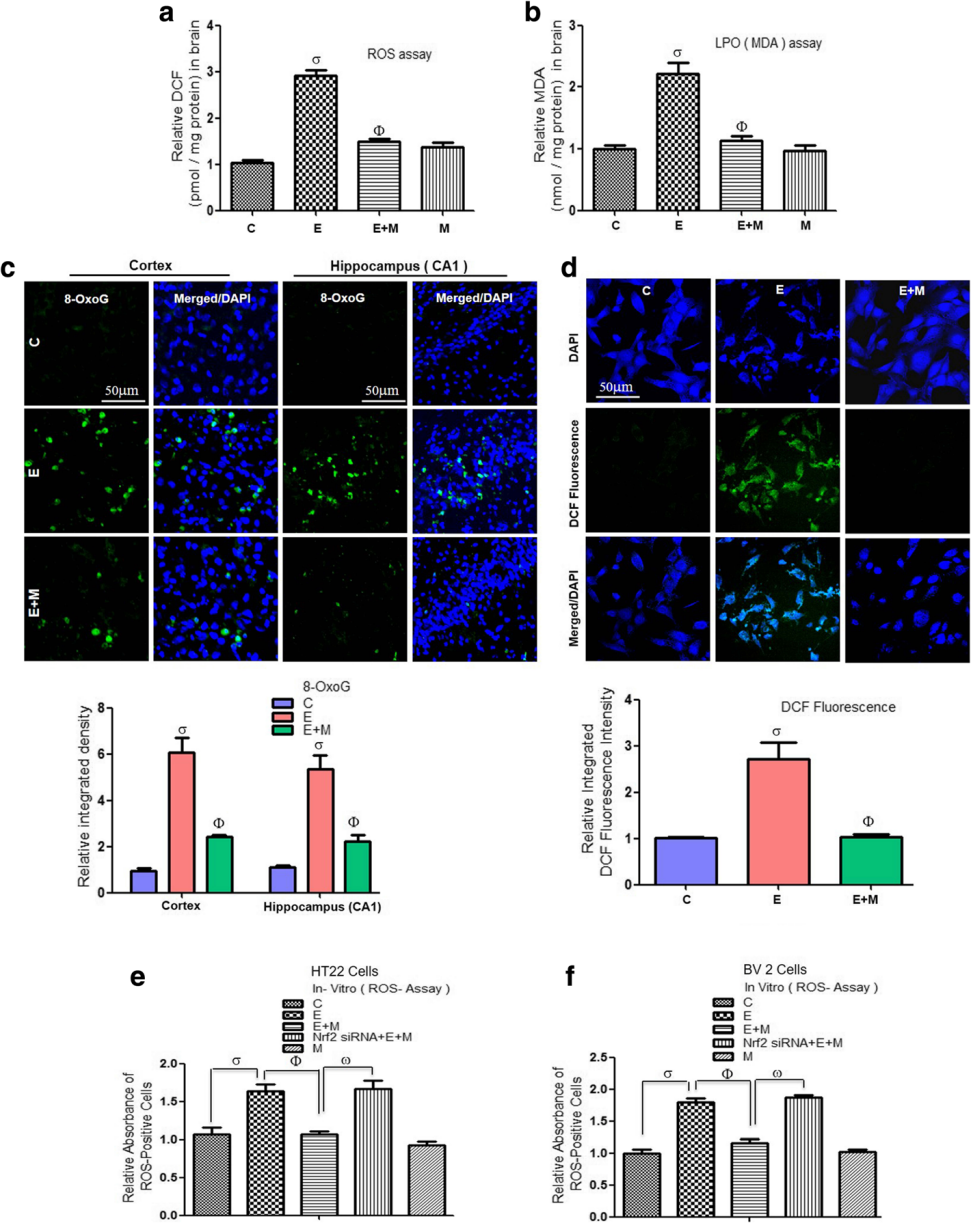
Acute melatonin attenuated the acute ethanol-induced increase in ROS and oxidative stress in the rat pups and in HT22 and BV2 cells. **a** A representative histogram of the ROS level in the brain homogenates of the rat pups. **b** A representative histogram of the MDA level in the brain homogenates of the rat pups. *n* = 10 pups/group, and the number of experiments = 3. **c** Representative image of immunofluorescence staining of 8-OxoG in the cortices and CA1 regions of the hippocampi in the rat pups. *n* = 5 pups/group, and the number of experiments = 3. Magnification × 40. Scale bar = 50 μm. **d** Representative images of the DCF immunofluorescence intensity in the HT22 cells. The number of experiments = 3. Magnification × 40. Scale bar = 50 μm. **e**, **f** A representative histogram of the relative absorbance of ROS-positive HT22 and BV2 cells respectively that were subjected to Nrf2 siRNA and treated with ethanol (100 mM) and melatonin (100 μM) for 12 h. The number of experiments = 3. The data are expressed as the mean ± SEM. The data are presented relative to control values. Significance = *P < 0.05.* σ Significantly different from the control saline-treated rat pups; Φ significantly different from the ethanol-treated rat pups. Similarly for in vitro studies, σ significantly different from the non-treated HT22 and BV2 cells, Φ significantly different from the ethanol-exposed HT22 and BV2 cells, and ω significantly different from the ethanol + melatonin-exposed HT22 and BV2 cells

To evaluate the melatonin antioxidant activity, we analyzed the ROS and LPO levels using ROS and MDA assays, respectively. Acute co-treatment of melatonin (20 mg/kg) with acute ethanol (5 g/kg) significantly alleviated the elevated ROS and LPO levels than those in rat pups that had been treated with ethanol alone (Fig. 2a, b). In addition, the results of 8-OxoG (an oxidative stress marker) labeling also revealed that acute co-treatment of melatonin significantly reduced the acute ethanol-induced, ROS-mediated oxidative stress, as shown by the decreased immunofluorescence reactivity of 8-OxoG in the developing rat brain (Fig. 2c). Additionally, melatonin (100 μM) treatment in vitro significantly reduced the elevated ROS production, as shown by the lower DCF fluorescence intensity in the ethanol (100 mM) exposed HT22 cells than in the HT22 cells that were exposed to ethanol alone (Fig. 2d). To determine the mechanism by which melatonin reduced the elevated ROS and oxidative stress, we used Nrf2 siRNA in the HT22 and BV2 cells. We observed that when we knocked down the Nrf2 genes with siRNA, melatonin (100 μM) was unable to reduce the elevated ROS in the ethanol (100 mM) exposed HT22 and BV2 cells respectively (Fig. 2e, f).

### Acute melatonin treatment upregulated Nrf2/HO-1/GCLM expression in the acute ethanol-treated rat pups and in HT22 and BV2 cells that were exposed to ethanol

**Fig. 3:**
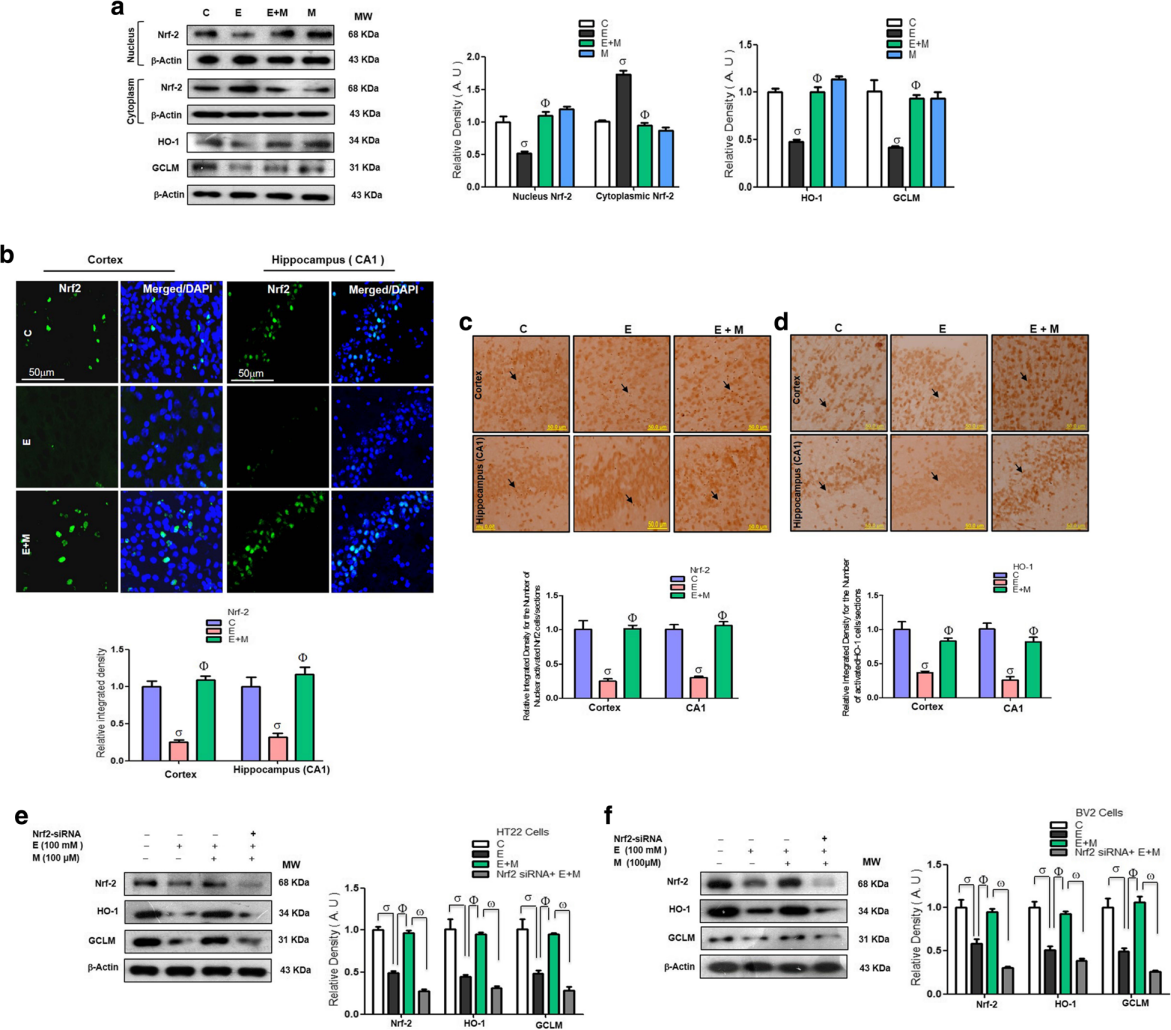
Acute melatonin treatment upregulated Nrf2/HO-1/GCLM expression in the acute ethanol-treated rat pups and in HT22 and BV2 cells that were exposed to ethanol. **a** Western blot analysis of nuclear/cytosolic Nrf2 expression using Nrf2, HO-1, and GCLM antibodies in the rat pups. The bands were quantified using Sigma Gel software, and the differences are presented in a histogram. β-Actin was used as a loading control. *n* = 10 pups/group, and the number of experiments = 3. **b** Representative immunofluorescence results of Nrf2 (FITC, DAPI, Blue) and **c**, **d** immunohistochemical results of Nrf2 and HO-1 respectively in the cortices and CA1 regions of the hippocampi in the rat pups. *n* = 5 pups/group, and the number of experiments = 3. Magnification × 20. Scale bar = 50 μm. **e**, **f** Western blots and the densitometric analysis of the Nrf2, HO-1, and GCLM expression in the HT22 and BV2 cells respectively that were subjected to Nrf2 siRNA and treated with ethanol (100 mM) and melatonin (100 μM) for 12 h. β-actin was used as a loading control. The data are expressed as the mean ± SEM, and the number of experiments = 3. The data are presented relative to control values. Significance = *P < 0.05.* σ Significantly different from the control saline-treated rat pups; Φ significantly different from the ethanol-treated rat pups. Similarly for in vitro studies, σ significantly different from the non-treated HT22 and BV2 cells; Φ significantly different from the ethanol-exposed HT22 and BV2 cells, and ω significantly different from the ethanol + melatonin-exposed HT22 and BV2 cells

Next, we observed that acute co-treatment of melatonin with ethanol reversed the effect of ethanol and significantly increased the expression of nuclear Nrf2 and its target genes HO-1 and GCLM compared with that in the rat pups that were treated with ethanol alone (Fig. 3a). The immunofluorescence and immunohistochemical results also indicated that melatonin remarkably increased immunohistochemical reactivity for the number of activated nuclear activated Nrf2 and HO-1 reactive cells in the cortices and hippocampi of the ethanol-treated rat pups (Fig. 3b–d). Further, we observed reduced expression of Nrf2 and its target genes HO-1 and GCLM in the ethanol (100 mM) exposed HT22 and BV2 cells, and this change was reversed by melatonin (100 μM) treatment, leading to a significant increase in the expression of Nrf2, HO-1, and GCLM. However, melatonin treatment did not increase the expression of Nrf2, HO-1, and GCLM in the ethanol-exposed HT22 and BV2 cells in which Nrf2 genes had been knocked down by Nrf2 siRNA (Fig.3e, f). These results revealed that melatonin attenuated the elevation in ROS and oxidative stress by upregulating the master endogenous antioxidant Nrf2.

### Acute melatonin prevents the acute ethanol-induced activation of the MAPK p-P38/p-JNK pathway in the rat pups and in HT22 cells that were exposed to ethanol

**Fig. 4:**
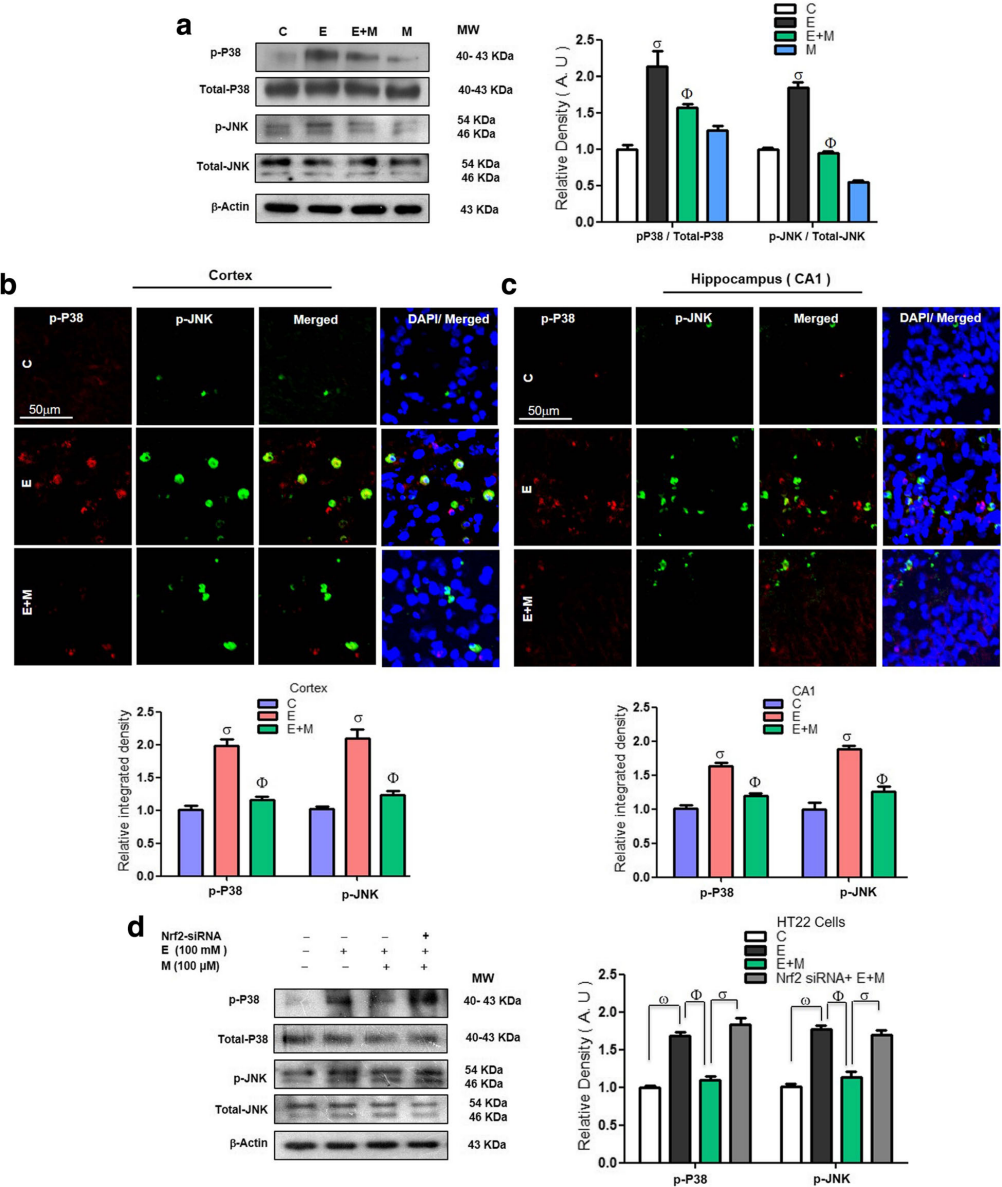
Acute melatonin prevents the acute ethanol-induced activation of the MAPK p-P38/p-JNK pathway in the rat pups and in HT22 cells that were exposed to ethanol. **a** Western blot results of p-P38, total P-38, p-JNK, and total JNK in the rat pups. The bands were quantified using Sigma Gel software, and the differences are presented in a histogram. β-Actin was used as a loading control. *n* = 10 pups/group, and the number of experiments = 3. **b**, **c** Representative images of the co-localized immunofluorescence reactivity of p-P38 and p-JNK in the cortices and CA1 regions of the hippocampi in the rat pups. *n* = 5 pups/group, and the number of experiments = 3. Magnification × 40. Scale bar = 50 μm. **d** Western blots and the densitometric analysis of p-P38, total P-38, p-JNK, and total JNK expression in the HT22 cells that were subjected to Nrf2 siRNA and treated with ethanol (100 mM) and melatonin (100 μM) for 12 h. β-Actin was used as a loading control. The data are expressed as the mean ± SEM, and the number of experiments = 3. The data are presented relative to control values. Significance = *P < 0.05.* σ Significantly different from the control saline-treated rat pups; Φ significantly different from the ethanol-treated rat pups. Similarly for in vitro studies, σ significantly different from the non-treated HT22 cells, Φ significantly different from the ethanol-exposed HT22 cells, and ω significantly different from the ethanol+melatonin-exposed HT22 cells

ROS-mediated oxidative stress activated the MAPK-p-P38/p-JNK pathway, a key junction between neuroinflammation and apoptotic neurodegeneration [30]. Ethanol-induced oxidative stress is mediated by activation of the p-P38-MAPK pathway in HT22 cells [31]. Herein, we observed that acute ethanol administration increased the expression of MAPK-p-P38/p-JNK pathway components compared with that in the control saline-treated rat pups. Acute co-treatment of melatonin with ethanol reversed the effect of ethanol and significantly reduced the expression of MAPK-p-P38/p-JNK pathway components compared to that in the rat pups that were treated with ethanol alone (Fig. 4a). The double immunofluorescence results also indicated that melatonin significantly reduced the co-localized immunofluorescence reactivity of MAPK-p-P38 and p-JNK in the cortices and hippocampi of the ethanol-treated rat pups compared to that in those of the rat pups that were treated with ethanol alone (Fig. 4b, c). We determined that melatonin (100 μM) could not reduce the ethanol-induced (100 mM) activation of the MAPK-p-P38/p-JNK pathway in the ethanol-exposed HT22 cells after the knockdown of Nrf2 genes by Nrf2 siRNA, which further confirmed the role of Nrf2 in this process (Fig. 4d).

### Acute melatonin reduced the level of activated gliosis in the acute ethanol-treated rat pups and activated microglia in the BV2 cells that were exposed to ethanol

**Fig. 5:**
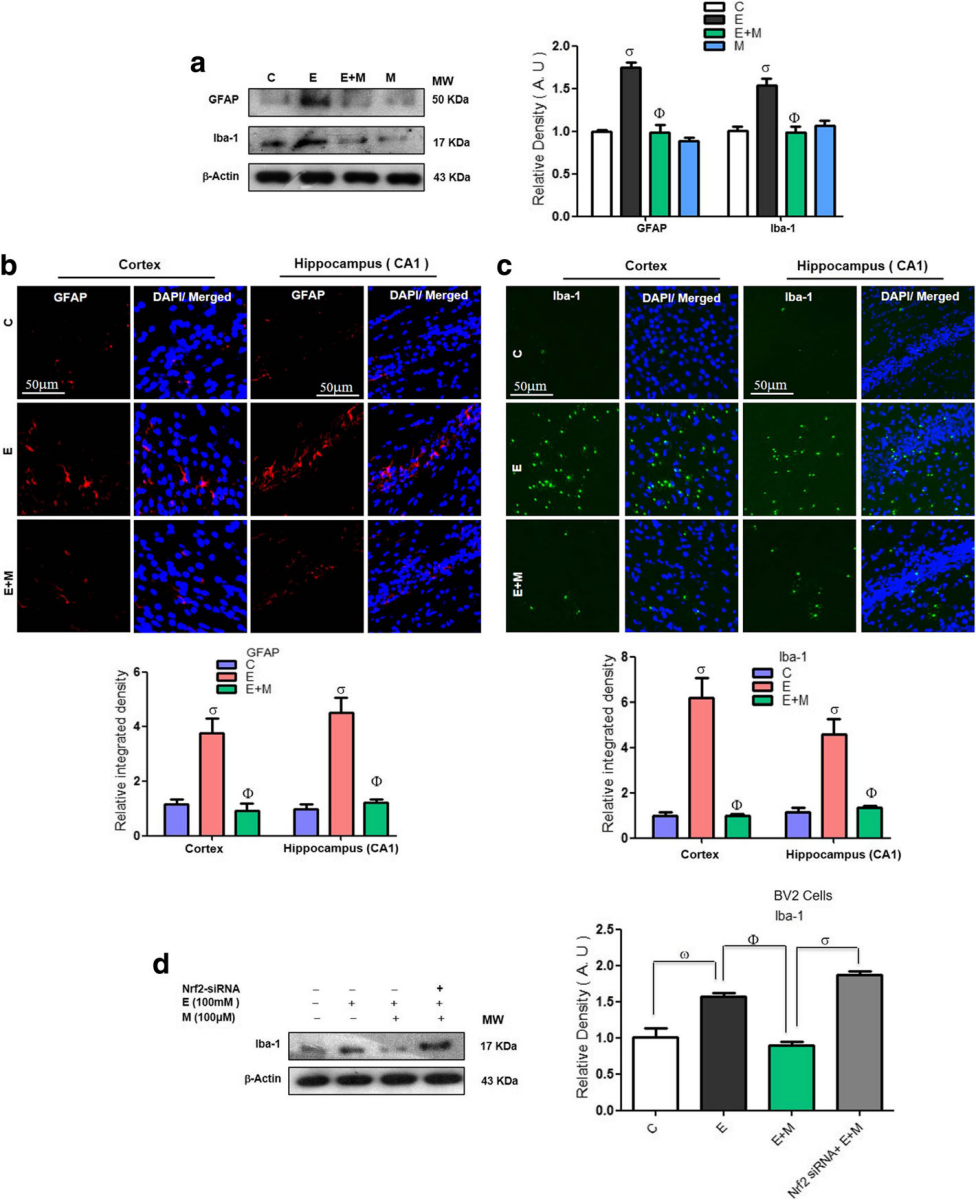
Acute melatonin reduced the level of activated gliosis in the acute ethanol-treated rat pups and activated microglia in the BV2 cells that were exposed to ethanol. **a** Western blot analysis of GFAP and Iba-1 in the rat pups. The bands were quantified using Sigma Gel software, and the differences are presented in a histogram. β-actin was used as a loading control. *n* = 10 pups/group, and the number of experiments = 3. **b**, **c** Representative images showing the immunofluorescence analysis of GFAP and Iba-1 in the cortices and the CA1 regions of the hippocampi in the rat pups, respectively. *n* = 5 pups/group, and the number of experiments = 3. Magnification × 40. Scale bar = 50 μm. **d** Western blots and the densitometric analysis of Iba-1 expression in the BV2 cells that were subjected to Nrf2 siRNA and treated with ethanol (100 mM) and melatonin (100 μM) for 12 h. β-Actin was used as a loading control. The number of experiments = 3. The data are expressed as the mean ± SEM. The data are presented relative to control values. Significance = *P < 0.05.* σ Significantly different from the control saline-treated rat pups; Φ significantly different from the ethanol-treated rat pups. Similarly for in vitro studies, σ significantly different from the non-treated BV2 cells; Φ significantly different from the ethanol-exposed BV2 cells, and ω significantly different from the ethanol + melatonin-exposed BV2 cells

Mounting literature reported that gliosis activation has been implicated in neuroinflammation and neurodegeneration [26]. Ethanol-induced, ROS-mediated oxidative stress triggers gliosis [32]. Our results indicated that ethanol remarkably activated gliosis, as evidenced by the significantly increased expression of GFAP (astrocytosis) and Iba-1 (activated microglia) compared to that in the control saline-treated rat pups. Recently, we and other authors have reported that both chronic and acute melatonin treatment prevented activated gliosis [26, 33]. In agreement with previous studies, the acute co-treatment of melatonin with ethanol in the current study significantly reduced the expression of both GFAP and Iba-1 compared to their expression in the ethanol alone-treated rat pups (Fig. 5a). The immunofluorescence results also demonstrated that acute melatonin treatment significantly decreased the immunofluorescence reactivity of GFAP and Iba-1 in the cortices and hippocampi of the ethanol-treated rat pups compared to that in those of the rat pups that were treated with ethanol alone (Fig. 5b, c). In addition to investigating the mechanistic role of Nrf2 in activated microglia, we knocked down Nrf2 via siRNA in BV2 cells. Western blot results demonstrated that when we knocked down the Nrf2 genes with siRNA, melatonin treatment (100 μM) was unable to reduce the elevated Iba-1 expression in the ethanol (100 mM) exposed BV2 cells (Fig. 5d). These in vivo and in vitro results confirmed that activation of Nrf2 has a key role in the attenuation of activated gliosis.

### Acute melatonin reduced the level of activated p-NF-_K_B/p-IKKβ in the acute ethanol-treated rat pups and in BV2 cells that were exposed to ethanol

**Fig. 6:**
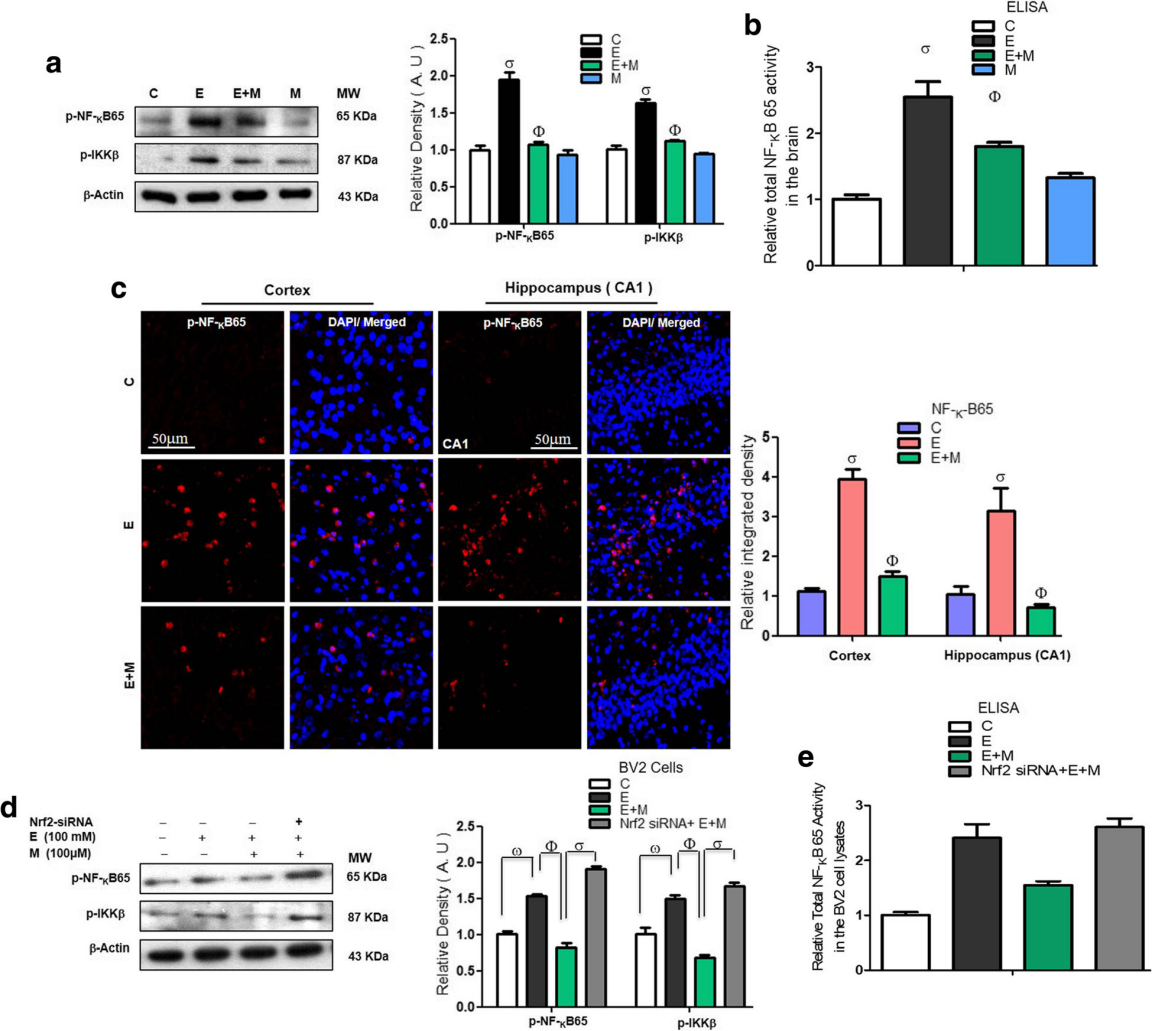
Acute melatonin reduced the level of activated p-NF-_K_B/p-IKKβ in the acute ethanol-treated rat pups and in BV2 cells that were exposed to ethanol. **a** Western blot analysis of p-NF-_K_B65 and p-IKKβ in the rat pups. The bands were quantified using Sigma Gel software, and the differences are presented in a histogram. β-Actin was used as a loading control. *n* = 10 pups/group, and the number of experiments = 3. **b** Representative histogram indicates the ELISA analysis of NF-_K_Bp65 level in the brain homogenates of the rat pups. *n* = 10 pups/group, and the number of experiments = 3. **c** Representative image of the p-NF-_K_B65 immunofluorescence reactivity in the cortices and CA1 regions of the hippocampi in the rat pups. *n* = 5 pups/group, and the number of experiments = 3. Magnification × 40, Scale bar = 50 μm. **d** Western blots and the densitometric analysis of the NF-_K_B and IKKβ expression levels in the BV2 cells that were subjected to Nrf2 siRNA and treated with ethanol (100 mM) and melatonin (100 μM) for 12 h. β-actin was used as a loading control. The data are expressed as the mean ± SEM. **e** A representative histogram indicates the ELISA analysis of NF-_K_Bp65 level in the BV2 cells that were subjected to Nrf2 siRNA and treated with ethanol (100 mM) and melatonin (100 μM) for 12 h. The number of experiments = 3. The data are expressed as the mean ± SEM. The data are presented relative to control values. Significance = *P < 0.05.* σ Significantly different from the control saline-treated rat pups; Φ significantly different from the ethanol-treated rat pups. Similarly for in vitro studies, σ significantly different from the non-treated BV2 cells; Φ significantly different from the ethanol-exposed BV2 cells, and ω significantly different from the ethanol+melatonin-exposed BV2 cells

The activated p-NF-_K_B/p-IKKβ pathway is the key mediator between neuroinflammation and neurodegeneration [34]. Elevated NF-_K_B expression has been observed in ethanol-treated pups [35]. In agreement with previous studies, we also observed an increased level of p-NF-_K_B/p-IKKβ in the ethanol-treated rat pups compared to that in the control saline-treated rat pups. Acute co-treatment of melatonin with the ethanol significantly attenuated the p-NF-_K_B/p-IKKβ levels compared with those in the ethanol alone-treated rat pups (Fig. 6a). Further, to confirm the western blot results for the NF-KB, we applied the ELISA on the homogenized brain tissue of all group. In parallel with western blot results ELISA results indicated that acute melatonin combat against acute ethanol-induced elevated NFKB activity (Fig. 6b). Next, immunofluorescence results also indicated that acute melatonin treatment significantly attenuated the p-NF_-K_B immunofluorescence reactivity in the cortices and hippocampi of the ethanol-treated rat pups compared to that in those of the ethanol alone-treated rat pups (Fig. 6c). Bundles good evidences reported that activated Nrf2 antagonize the NF-_K_B activity and vice versa activated NF-_K_B antagonized the Nrf2 activity. Here in order to know the correlation of these two markers, we applied the immunoblot and ELISA on the cell lysates of the treated BV2 cells exposed to Nrf2 siRNA. Immunoblot results revealed and confirmed that when we knocked down the Nrf2 genes via siRNA, melatonin (100 μM) treatment did not reduce the elevated expression of p-NF-_K_B/p-IKKβ in the ethanol (100 mM) treated BV2 cells (Fig. 6d). The ELISA results also notated that ethanol-induced activated NF-_K_B prevented by melatonin, nevertheless the Nrf2 siRNA abolished the activity of melatonin and could reduce the over activity of NFKB in the ethanol-exposed BV2 cells subjected to Nrf2 siRNA (Fig. 6e). All these results suggested that melatonin via Nrf2 dependently inhibit the main marker of neuroinflammation and neurodegeneration.

### Acute melatonin attenuated the neuroinflammation in the acute ethanol-treated rat pups and in BV2 cells that were exposed to ethanol

**Fig. 7:**
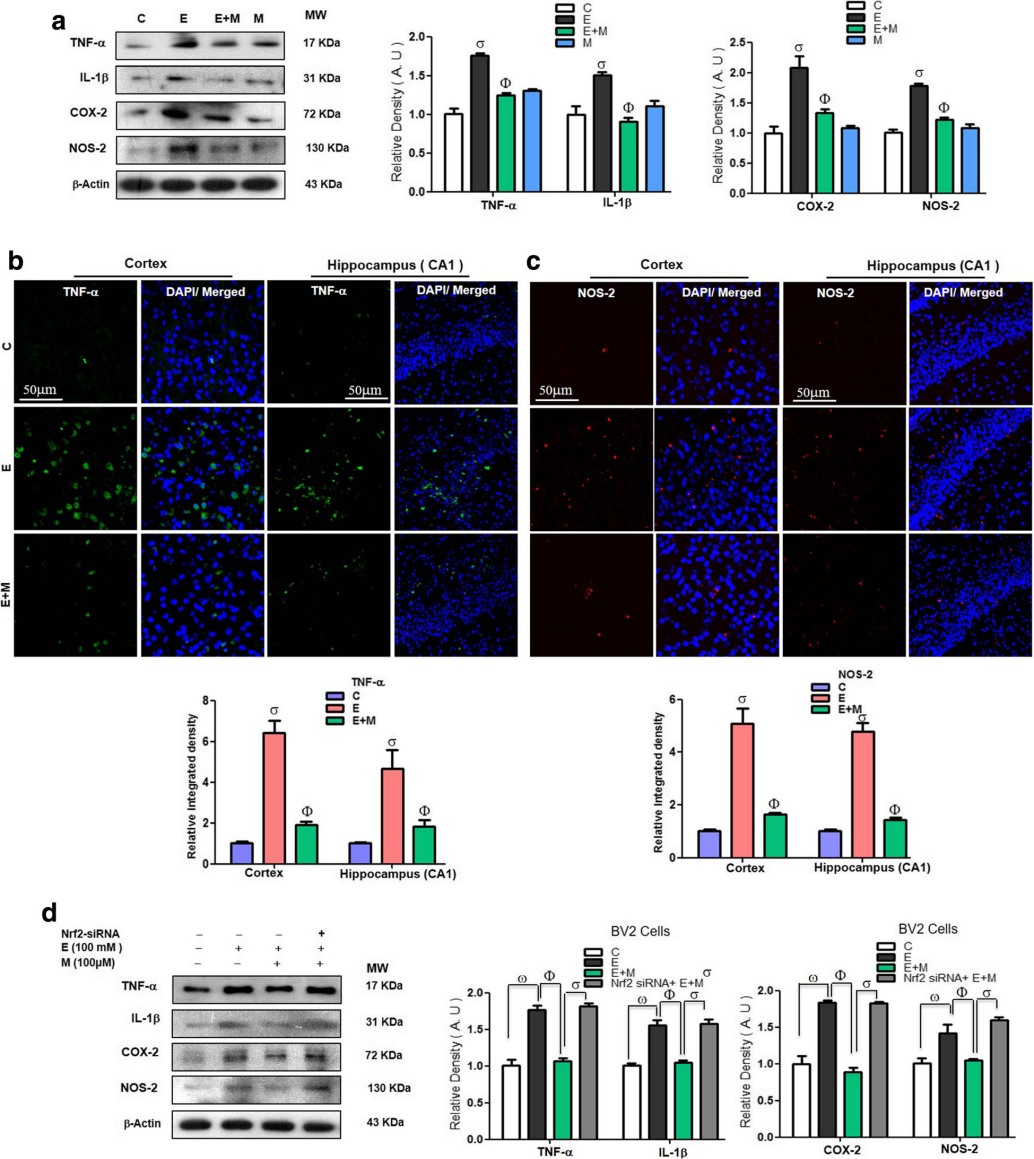
Acute melatonin attenuated the various inflammatory mediators in the acute ethanol-treated rat pups and in BV2 cells that were exposed to ethanol. **a** The western blot analysis of TNF-α, IL-1β, COX-2, and NOS-2 in the rat pups. The bands were quantified using Sigma Gel software, and the differences are represented by a histogram. β-Actin was used as a loading control. *n* = 10 pups/group, and the number of experiment = 3. **b**, **c** The representative image shows immunofluorescence reactivity of TNF-α and NOS-2 in the cortices and CA1 region of the hippocampi in the rat pups. *n* = 5 pups/group, and the number of experiments = 3. Magnification × 40, scale bar = 50 μm. **d** Western blots and the densitometric analysis of TNF-α, IL-1β, COX-2, and NOS-2 expression levels in the BV2 cells that were subjected to Nrf2 siRNA and treated with ethanol (100 mM) and melatonin (100 μM) for 12 h. β-Actin was used as a loading control. The number of experiments = 3. The data are expressed as the mean ± SEM. The data are presented relative to control values. Significance = *P < 0.05.* σ Significantly different from the control saline-treated rat pups; Φ significantly different from the ethanol-treated rat pups. Similarly for in vitro studies, σ significantly different from the non-treated BV2 cells; Φ significantly different from the ethanol-exposed BV2 cells, and ω significantly different from the ethanol+melatonin-exposed BV2 cells

Acute ethanol exposure incites the activation of various inflammatory mediators [35]. We also observed that acute ethanol exposure increased the expression levels of several key inflammatory markers, such as TNF-α, IL-1β, COX-2, and NOS-2, compared to those in the control saline-treated pups. Acute co-treatment of melatonin with ethanol reversed the effect of ethanol and significantly reduced the expression levels of these inflammatory markers compared with those in the ethanol alone-treated rat pups (Fig. 7a). Immunofluorescence results demonstrated that acute melatonin exposure prevented the immunofluorescence reactivity of TNF-α and NOS-2 in the cortices and the CA1 regions of the hippocampi in the ethanol-treated rat pups compared to that in the ethanol alone-treated rat pups (Fig. 7b, c). Further, we observed via western blots that when we knocked down the expression of Nrf2 in BV2 cells with Nrf2 siRNA, melatonin (100 μM) treatment was unable to attenuate the ethanol-induced elevation in TNF-α, IL-1β, COX-2, and NOS-2 expression in the BV2 cells (Fig. 7d), which indicates that the anti-oxidant Nrf2 is a key in the regulation of inflammatory mediators and prevents the neuroinflammation.

### Acute melatonin attenuated the acute ethanol-induced apoptosis and neurodegeneration in vivo and in vitro ethanol-exposed HT22 neuronal cells

**Fig. 8:**
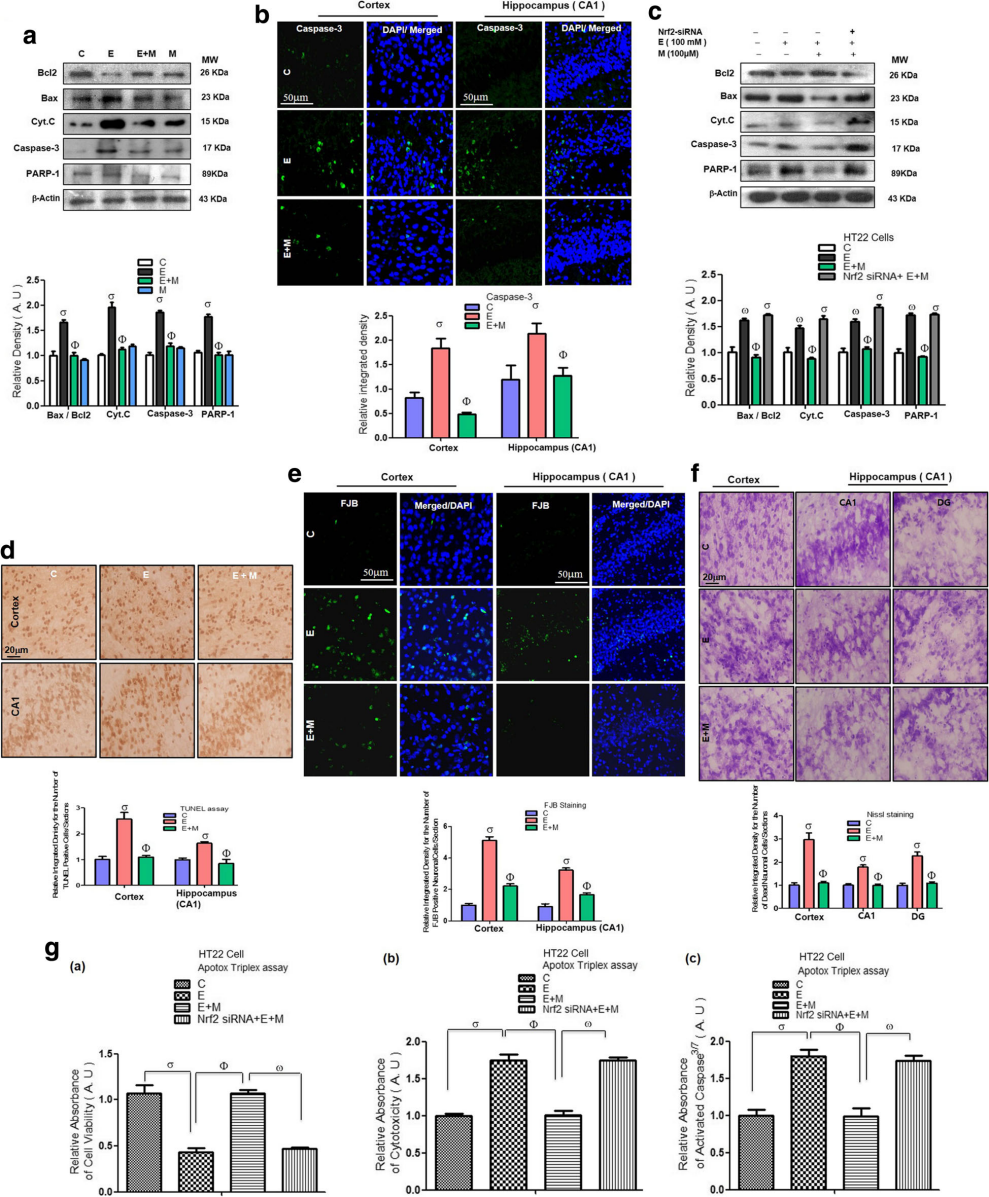
Acute melatonin attenuated the acute ethanol-induced apoptosis and neurodegeneration in vivo and in vitro ethanol-exposed HT22 neuronal cells. **a** Western blots analysis of Bax, Bcl2, Cyt.c, activated caspase-3 and PARP-1 antibodies. The bands were quantified using Sigma Gel software, and the differences are represented by a histogram. β-Actin was used as a loading control. *n* = 10 pups/group, and the number of experiments = 3. **b** Western blots and the densitometric analysis of Bax, Bcl2, Cyt.c, activated caspase-3 and PARP-1 expression levels in the HT22 cells that were subjected to Nrf2 siRNA and treated with ethanol (100 mM) and melatonin (100 μM) for 12 h. β-Actin was used as a loading control. The number of experiments = 3. **c** The representative image shows immunofluorescence reactivity of activated caspase-3 in the cortices and CA1 regions of the hippocampi in the rat pups. *n* = 5 pups/group, and the number of experiments = 3. Magnification × 40. Scale bar = 50 μm. **d** Shown images indicate the TUNEL immunohistochemical staining in the cortices and CA1 regions of the hippocampi in the rat pups. *n* = 5 pups/group, and the number of experiments = 3. Magnification × 20. Scale bar = 20 μm. **e** Shown images indicate the FJB immunohistochemical staining in the cortices and CA1 regions of the hippocampi in the rat pups. *n* = 5 pups/group, and the number of experiments = 3. Magnification × 40. Scale bar = 50 μm. **f** Representative photomicrograph of Nissl staining in the cortices; and DG and CA1 regions of the hippocampi in the rat pups. *n* = 5 pups/group, and the number of experiments = 3. Magnification × 20. Scale bar = 20 μm. **g** Apo-Tox Glo™ assay in the neuronal HT22 cells using Nrf2 siRNA. (a–c) The cell viability, cytotoxicity and activation of caspase-3/7 respectively in the HT22 cells that were subjected to Nrf2 siRNA and treated with ethanol (100 mM) and melatonin (100 μM) for 12 h. The number of experiments = 3. The data are expressed as the mean ± SEM. The data are presented relative to control values. Significance = *P < 0.05.* σ Significantly different from the control saline-treated rat pups; Φ significantly different from the ethanol-treated rat pups. Similarly for in vitro studies, σ significantly different from the non-treated HT22 cell; Φ significantly different from the ethanol-exposed HT22 cells and ω significantly different from the ethanol + melatonin-exposed HT22 cells

Next, we observed that ethanol decreased the anti-apoptotic Bcl2 expression and increased the expression of apoptotic Bax; thus, the Bax/Bcl2 ratio was increased in the ethanol-treated rat pups compared with that in the control saline-treated rat pups. Acute co-treatment of melatonin with ethanol reversed the effect of ethanol and significantly reduced the Bax/Bcl2 ratio compared with that in the ethanol alone-treated rat pups (Fig. 8a). Similarly, we observed that acute co-treatment of melatonin with ethanol reversed the effect of ethanol and significantly reduced the Cyt.c and activation of caspase-3 expression compared with that in the ethanol alone-treated rat pups (Fig. 8a). Moreover, immunofluorescence results also indicated that acute co-treatment of melatonin with ethanol reversed the effect of ethanol and significantly reduced the immunofluorescence reactivity of activated caspase-3 in the cortices and hippocampi compared with that in those of the ethanol alone-treated rat pups (Fig. 8b). Next, we observed that acute co-treatment of melatonin with ethanol reversed the effect of ethanol and significantly decreased the level of cleaved PARP-1 compared with that in the ethanol alone-treated rat pups (Fig. 8a). To investigate the role of the Nrf2/HO-1 pathway in the ethanol-induced apoptotic neurodegeneration, we assessed the western blot in the neuronal HT22 cells that had been subjected with Nrf2 siRNA and exposed to ethanol (100 mM) and melatonin (100 μM). Western blot results indicated that when we knocked down the expression of Nrf2 in HT22 cells with Nrf2 siRNA, melatonin treatment was unable to attenuate the ethanol-induced elevation in apoptotic markers such as Bax/Bcl2 ratio, Cyt.c, cleaved caspase-3, and PARP-1 in the HT22 cells (Fig. 8c), which indicates that the Nrf2 has a pivotal and promising role in the attenuation of apoptotic neurodegeneration.

TUNEL and FJB staining results showed a significant increase in the immunohistochemical reactivity for the number of activated TUNEL-positive neuronal cells nuclei and number of FJB-positive neuronal cells in the ethanol-treated rat pups compared with that in the control saline-treated rat pups. Acute co-treatment of melatonin with ethanol remarkably attenuated the effect of ethanol and significantly reduced the immunohistochemical reactivity for number of activated TUNEL-positive neuronal cells nuclei and FJB-positive neuronal cells compared with that in the ethanol alone-treated rat pups (Fig. 8d, e). In accordance with the TUNEL and FJB results, the Nissl staining results also showed that after ethanol administration, immunohistochemical reactivity for the number of apoptotic and degenerated neurons (damaged, fragmented, or shrunken neuronal cells) was significantly higher in the ethanol-treated rat pups than in the control saline-treated rat-pups (Fig. 8d). The administration of acute co-treatment of melatonin with ethanol reversed the effect of ethanol and significantly reduced immunohistochemical reactivity for the number of degenerated neuronal cells compared with that in the ethanol alone-treated rat pups (Fig. 8f). These results showed that melatonin prevented ethanol-induced apoptotic neurodegeneration in the developing rat brain. To determine the role of the antioxidant Nrf2 pathway in ethanol-induced neurotoxicity, we performed the Apo-Tox Glo™ triplex assay in the neuronal HT22 cells that had been treated with Nrf2 siRNA. The result showed that 100 μM melatonin reversed the effect of ethanol (100 mM), significantly increased the cell viability and reduced the cytotoxicity and the activation of caspase-3/7 in the HT22 cells (Fig. 8g (a–c)). However, after the cells were exposed to Nrf2 siRNA, melatonin (100 μM) was not effective against ethanol-induced neurotoxicity and could not increase the cell viability, reduce the cytotoxicity or reverse the activation of caspase-3/7 in the HT22 cells (Fig. 8g (a–c)). These in vitro results suggested that melatonin acts as a potent antioxidant in an Nrf2-dependent manner to reduce ethanol-induced, ROS-mediated neurotoxicity.

## Discussion

The objective of this study was to determine the underlying potential antioxidant neuroprotective mechanism of the acute treatment of melatonin against the acute ethanol-induced elevated ROS, neuroinflammation, apoptosis, and neurodegeneration in the postnatal rat brain. The brain is one of the most vulnerable organs to oxidative stress due to the high oxygen demand, the presence of redox-active metals, and the comparatively low levels of endogenous antioxidant genes, which play a key role in preventing and eliminating elevated ROS. The accumulation of elevated ROS triggers oxidative stress, which consequently leads to detrimental effects and plays a key role in the neuropathology of various neurological disorders [7–9]. The endogenous antioxidant system, particularly Nrf2 and its target genes HO-1 and GCLM, has been implicated in the prevention of elevated ROS-induced oxidative stress and the consequences of ROS-induced oxidative stress. The activation of the Nrf2 pathway via antioxidants, particularly endogenous compounds such as melatonin, is a compelling approach for inducing endogenous antioxidant production to combat the elevated ROS and oxidative stress. Many studies have reported that melatonin activates the translocation of Nrf2 from the cytosol to the nucleus, where it activates antioxidative enzymes, such as HO-1 and NAPDH quinine dehydrogenase-1 (NQO1), and produces a notable antioxidative response to counteract elevated ROS and oxidative stress [36–39]. Further studies have also demonstrated that melatonin induced the increased expression of nuclear Nrf2- and HO-1-induced neuroprotection against various CNS insults [37, 40–42]. Ethanol exposure generated and elevated ROS, which led to the neuronal degeneration [2, 5, 6]. Herein, we observed elevated ROS and oxidative stress in both in vivo and in vitro models that were exposed to ethanol; this change might be implicated in the detrimental effects of ethanol exposure in the developing brain. Moreover, we also noticed that ethanol exposure diminished the antioxidant Nrf2/HO-1 mechanism. When we acutely co-administered melatonin with the ethanol-exposed rat pups and co-treated melatonin with ethanol-exposed HT22 and BV2 cells, the endogenous Nrf2/HO-1 mechanism stimulated which consequently alleviated elevated ROS and oxidative stress in both in vitro and in vivo studies (Figs. 2a–f and 3a–e).

Ethanol exposure, directly or indirectly through excessive ROS and oxidative stress, results in the overexpression and activation of the p-JNK/p38MAPK pathway. Activated and impaired MAPK signaling has been implicated in various neurological disorders in animal and human models of neurological disorders. Within the MAPK signaling pathway, p-JNK and p-P38 signaling is initiated by CNS insults directly or indirectly in response to oxidative stress [43–46]. P38 MAPK inhibitors have been identified as promising therapeutic options for neurodegeneration [47]. Han et al. observed that ethanol-activated p-JNK and suppressed survival signaling, thus leading to cell death and apoptosis in the developing rat brain [48]. Consistent with the previous studies, we also found that ethanol exposure activated p-JNK/P38 MAPK signaling in the developing rat brain and in ethanol-exposed HT22 cells. Moreover, acute melatonin treatment alleviated the effects of ethanol on MAPK and significantly reduced p-P38 and p-JNK expression in the postnatal rat brain and in ethanol-exposed HT22 cells (Fig. 4a–d).

A recent study indicated that melatonin treatment prevented the activation of gliosis and other inflammatory mediators, such as TNF-α and COX-2 [34]. Ethanol exposure may have mediated neuroinflammation by activating astrocytes and microglia [32, 49]. It has been reported that activated glial and neuronal cells lead to the activation of stress kinases, such as p-IKKβ/p-NF_K_B, which contribute to the activation of various inflammatory mediators to induce neuroinflammation [34, 50]. Ethanol-induced activation of the cytokines and chemokines lead to the neuroinflammation [29]. Melatonin is a remarkable, versatile pleiotropic agent with potent antioxidant, anti-inflammatory, and anti-apoptotic effects [51]. Recently, the reduction of neonatal neuroinflammation via treatment with melatonin was investigated [20]. Melatonin increased Nrf2 expression, which resulted in attenuated oxidative stress and consequently reduced neuroinflammation by decreasing the expression of NF-_K_B/IKKβ pathway components and other inflammatory mediators (TNF-α, IL-1β, NOS-2 and COX-2) [52]. The results of our current study also indicated that treatment with melatonin activated the Nrf2-dependent antioxidant mechanism and attenuated ethanol-induced neuroinflammation by reducing the activation of gliosis, NF-_K_B/IKKβ expression and the expression of other neuroinflammatory mediators, such as TNF-α, IL-1β, NOS-2 and COX-2 in the postnatal rat brain and in ethanol-exposed BV2 cells (Fig. 5a–d, 6a–c, and 7a–d).

It has been reported that Nrf2 pathway activation induced neuroprotection [53–56]. Ethanol-induced, ROS-mediated apoptotic neurodegeneration occurs through the upregulation of various apoptotic markers and the downregulation of anti-apoptotic markers. Ethanol triggered apoptosis in neurons via the downregulation of Bcl2 and the overexpression of Bax and disturbed the mitochondrial homeostasis and the activation and release of Cyt.c, which led to the activation of caspase-3 and cleaved PARP-1; these events led to apoptotic neurodegeneration [1, 2, 29]. Melatonin has been shown to play an important role in mitochondrial homeostasis and suppresses apoptosis by upregulating the anti-apoptotic proteins of the Bcl2 family and downregulating apoptotic Bax proteins; this effect prevents Cyt.c release and the activation of caspase-3, thereby preventing apoptosis and neurodegeneration [57]. In the current study, we observed that acute melatonin treatment prevented apoptosis in the developing rat brain and in ethanol-exposed HT22 cells through the suppression of Bax/Bcl2, Cyt.c, activated caspase-3, and cleaved PARP-1 expression (Fig. 8a–c). In addition, the results of the activated caspase-3 immunofluorescence reactivity, TUNEL, FJB and Nissl immunohistochemical staining assays indicated that melatonin prevented apoptosis and neurodegeneration in the ethanol-treated rat pups (Fig. 8d–f). Furthermore, we also elucidated the mechanism by demonstrating that melatonin prevented ethanol-induced neurotoxicity in vitro in HT22 cells in an Nrf2-dependent manner because when we knocked down Nrf2 with Nrf2 siRNA in HT22 cells, melatonin was ineffective (Fig. 8g (a–c)). This finding further indicated that the ROS-induced oxidative stress was attenuated by the activation of the endogenous antioxidant Nrf2/HO-1 by melatonin treatment and that melatonin plays a key role in the prevention of apoptosis and neurodegeneration in the developing brain (Fig. 8a–g).

## Conclusions

In conclusion, collectively, our in vivo and in vitro findings demonstrated that acute melatonin acted as a potent endogenous antioxidant neuroprotective neurohormone that stimulated the master endogenous antioxidant Nrf2 and ameliorated acute ethanol-induced elevated ROS, neuroinflammation, and neurodegeneration in the developing rat brain. We propose that acute melatonin halts acute ethanol-induced detrimental effects and could be beneficial to prevent neurotoxicity in fetal alcohol syndrome.

## Abbreviations

DAB: 3: 3′-Diaminobenzidine tetra hydrochloride
IL-1β: Interleukin-1β
TRITC: Tetramethyl rhodamine isothiocyanate
8-OxoG: 8-Oxoguanine
CNS: Central nervous system
COX2: Cyclooxygenase-2
Cyt.c: Cytochrome c
DAPI: 4′, 6′-Diamidino-2-phenylindole
DCF: 2′7′-Dichlorofluorescei
DCFH-DA: 2′7′-Dichlorodihydrofluorescein diacetate
DMEM: Dulbecco’s modified Eagle medium
DMSO: Dimethyl sulfoxide
FAS: Fetal alcohol syndrome
FBS: Fetal bovine serum
FITC: Fluorescein isothiocyanate
FJB: Fluoro-jade B
GCLM: Glutathione cysteine ligase modulatory subunit
GFAP: Glial fibrillary acidic protein
HO-1: Heme oxygenase-1
HRP: Horseradish peroxidase
LPO: Lipid peroxidation
MAPKp-P38: Mitogen activated protein kinase phosphorylated-P38
MDA: Malondialdehyde
NOS2: Nitric oxide synthase-2
Nrf2: Nuclear factor erythroid 2-related factor 2
PARP-1: Poly (ADP-ribose) polymerase-1
P-JNK: Phospho-c-Jun N-terminal kinase 1
ROS: Reactive oxygen species
siRNA: small interfering RNA
TEP: 1, 1, 3, 3- Tetra ethoxy propane
TIF: Tagged image file format
TUNEL: Terminal deoxynucleotidyl transferase dUTP nick end labeling.
